# Combining reverse Monte Carlo analysis of X-ray scattering and extended X-ray absorption fine structure spectra of very small nanoparticles

**DOI:** 10.1107/S1600576722010858

**Published:** 2023-02-01

**Authors:** Markus Winterer, Jeremias Geiß

**Affiliations:** a Nanoparticle Process Technology and CENIDE (Center for Nanointegration Duisburg-Essen), University of Duisburg-Essen, Lotharstrasse 1, 47057 Duisberg, Germany; Argonne National Laboratory, USA

**Keywords:** extended X-ray absorption fine structure, EXAFS, wide-angle X-ray scattering, WAXS, reverse Monte Carlo, RMC, nanocrystals, LaFeO_3_

## Abstract

Finite size effects in partial pair distribution functions generate artefacts in the scattering structure factor and scattering intensity. It is shown how they can be overcome using a binned version of the Debye scattering equation. Accordingly, reverse Monte Carlo simulations are used for very small nanoparticles of LaFeO_3_ with diameters below 10 nm to simultaneously analyse X-ray scattering data and extended X-ray absorption fine structure spectra at the La *K* and Fe *K* edges. The structural information obtained is consistent regarding local structure and long-range order.

## Abstract 2

Computing scattering intensity using the Debye scattering equation after binning interatomic distances avoids finite size artefacts and is efficient enough for simultaneous refinement of scattering data and extended X-ray absorption spectra by reverse Monte Carlo simulations.

## 1. Introduction

It is well known that size, structure and properties are closely related for nanoscaled materials. When particles – here crystallites – become very small, *i.e.* have diameters below about 10 nm, a significant fraction of atoms are located at the particle surface. In the case of crystalline particles, the translational symmetry is broken, the diffraction patterns are broadened and structural information is lost as disorder is introduced heterogeneously. However, spectroscopic data, especially data from X-ray absorption spectroscopy, contain structural information which is not dependent on translational order, *i.e.* local structure. In this contribution, we will use the complex oxide LaFeO_3_ as a model system to extract structural information which is consistent regarding both structural hierarchies, short- and long-range order.

Different approaches may be used to analyse scattering and extended X-ray absorption fine structure (EXAFS) data simultaneously. Algorithms are based on (i) variation of crystallographic parameters similar to Rietveld refinements, (ii) periodic (unit-cell-based) models computing the structure factor for the scattering data, (iii) periodic models computing the total pair distribution function (PDF) for the scattering data and (iv) cluster (non-periodic) models.

The use of the scattering structure factor in algorithm (ii) has the advantage of being close to the ‘raw’ scattering data, whereas approach (iii) circumvents problems of finite size in the structural model as well as in the experimental data. Finite size effects are observable for very small scale nanomaterials, *i.e.* sizes smaller than 10 nm (see *e.g.* Page *et al.*, 2004 ▸; Gilbert, 2008 ▸).

Binsted *et al.* (1995 ▸, 1996 ▸) developed an approach using algorithm (i) to combine EXAFS and powder diffraction analysis by refinement of a crystallographic model including point symmetry around the EXAFS absorber atoms through Rietveld-type parameters (see also Binsted *et al.*, 1998 ▸, 2001 ▸; Weller *et al.*, 1999 ▸).

In an approach corresponding to algorithm (ii), Wicks & McGreevy (1995 ▸) developed a reverse Monte Carlo (RMC) method to simultaneously analyse neutron and X-ray structure factors along with EXAFS spectra (see also Winterer *et al.*, 2002 ▸; Arai *et al.*, 2007 ▸; Jóvári *et al.*, 2007 ▸, 2017 ▸; Gereben *et al.*, 2007 ▸; Kaban *et al.*, 2007 ▸). Mellergård & McGreevy (1999 ▸) used an intricate hybrid algorithm separating Bragg and diffuse scattering in RMC analysis of diffraction which was compared with algorithm (ii) by Sánchez-Gil *et al.* (2015 ▸).

Krayzman *et al.* (2008 ▸) extended an existing RMC code to simultaneously analyse EXAFS and total scattering PDFs [algorithm (iii)] (see also Krayzman *et al.*, 2009 ▸; Krayzman & Levin, 2010 ▸; Németh *et al.*, 2012 ▸).

The Debye scattering equation (DSE) (Debye, 1915 ▸) is an approach to directly compute scattering intensities from real-space (atomistic) models. Therefore, it is a natural interface between atomistic computer simulations and simulations of experimental data (Derlet *et al.*, 2004 ▸), and is the basis for algorithm (iv). Probably, the first application of the DSE to nanoparticles was the computation of electron scattering curves of small copper crystals by Germer & White (1941 ▸). For small, finite objects such as nanoparticles, the DSE is the appropriate method to compute the scattering intensity (Scardi *et al.*, 2016 ▸). Murray *et al.* (1993 ▸) simulated X-ray diffraction data of small CdSe nanocrystals (1–12 nm) applying the DSE. They used the algorithm of Hall & Monot (1991 ▸) to efficiently compute the DSE by binning interatomic distances. Belyakova *et al.* (2004 ▸) compared X-ray diffractograms and EXAFS spectra of Pd nanoparticles and MoS_2_ nanocomposites with simulations based on cluster models using the DSE. Derlet *et al.* (2004 ▸) developed a computationally efficient method to compute the DSE for up to 10^7^ atoms. Markmann *et al.* (2008 ▸) computed the DSE using a histogram of interatomic distances from atomic configurations. Beyerlein *et al.* (2010 ▸) used the DSE to simulate small-angle X-ray scattering and wide-angle X-ray scattering (WAXS) data, including the size distribution, extended defects and orientation of gold nanoparticles. Beyerlein (2013 ▸) described the general applicability of the DSE and its connection to atomistic computer simulations. The application of the DSE for data analysis has so far mostly been limited by the steep increase in computational expense with increasing number of atoms. Recently, Bertolotti *et al.* (2020 ▸) used the DSE to analyse WAXS data with regard to the size and shape of TiO_2_ nanoparticles.

In this contribution, we describe a novel implementation of algorithm (iv) to simultaneously analyse EXAFS spectra and X-ray scattering data by incorporation of the DSE into RMC simulation analysis (Winterer, 2000 ▸, 2002 ▸) of very small nanoparticles. We apply the method to La and Fe *K*-edge EXAFS spectra and WAXS data of nanocrystalline LaFeO_3_ particles using a cluster model. In general, the method can include X-ray, electron or neutron scattering data.

## 2. Theoretical background

RMC simulations are based on the Metropolis Monte Carlo (MC) algorithm, where the interatomic potential is replaced by the difference between experimental data, *i.e.* scattering intensity and/or EXAFS spectra, and simulations based on an atomic configuration (McGreevy & Pusztai, 1988 ▸). EXAFS spectra may be computed from partial pair distribution functions [pPDFs, *g*_*ij*_(*r*)] by integration over the product of the pPDFs and the EXAFS kernel γ_*ij*_(*k*, *r*) for the corresponding absorber–scatterer pair *ij* (Filipponi, 1994 ▸):

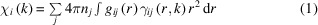

with

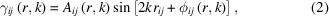

where *k* is the modulus of the wavevector of the photoelectron and *r* the interatomic distance (see supporting information). The EXAFS amplitude *A*(*r*, *k*) and phase ϕ(*r*, *k*) functions are taken from *ab initio* *FEFF* simulations (Rehr *et al.*, 2010 ▸) using the initial atom configuration (see Fig. S1 in the supporting information). The pPDFs,

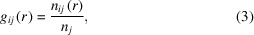

are defined by the number of atoms *j* at a distance *r* from atom *i* divided by the average number density of the neighbouring atom *j*,

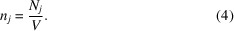




For isotropic samples, we may also use the pPDFs to compute the scattering intensity (see *e.g.* Cusack, 1987 ▸),

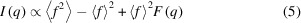

(*q* is the magnitude of the scattering vector; see supporting information), via the total structure factor

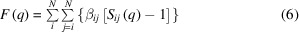

using the atomic form factors *f*, the coefficient β_*ij*_ (see section S2 in the supporting information) and the partial structure factors,

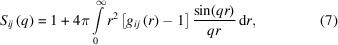

by integration over the product of [*g_ij_* (*r*) − 1] and the sinc function of *q* *r*.

Obviously, the pPDFs are the key element in RMC. They contain the relevant quantitative structural information regarding (*a*) coordination number (proportional to the area under a peak, zeroth moment of the distribution), (*b*) mean coordination distance (position of a peak, first moment) and (*c*) mean-square displacement (obtained from the second moment which is equivalent to the Debye–Waller factor in normal EXAFS analysis as a measure of the width of a peak).

The moment analysis of the pPDFs (Table 2) is the equivalent to a full (standard) EXAFS analysis (see *e.g.* Djenadic *et al.*, 2010 ▸). The advantage of a moment analysis of the pPDFs is that no Gaussian (or any other) distribution function is assumed and that higher moments (skewness and curtosis corresponding to the third and fourth cumulant) are available. However, often they are not significant.

In principle, we can use a mutual physical model to compute EXAFS spectra and X-ray scattering data as described. However, in the derivation of this structure factor *S_ij_* (*q*), it is assumed that the system is infinitely large, which is certainly not a good model for small nanoparticles. This assumption is used to separate the forward scattering and results in the term [*g_ij_* (*r*) − 1] in the (partial) structure factor (see *e.g.* Cusack, 1987 ▸).

## 3. Methodology, results and discussion

**Figure 1:**
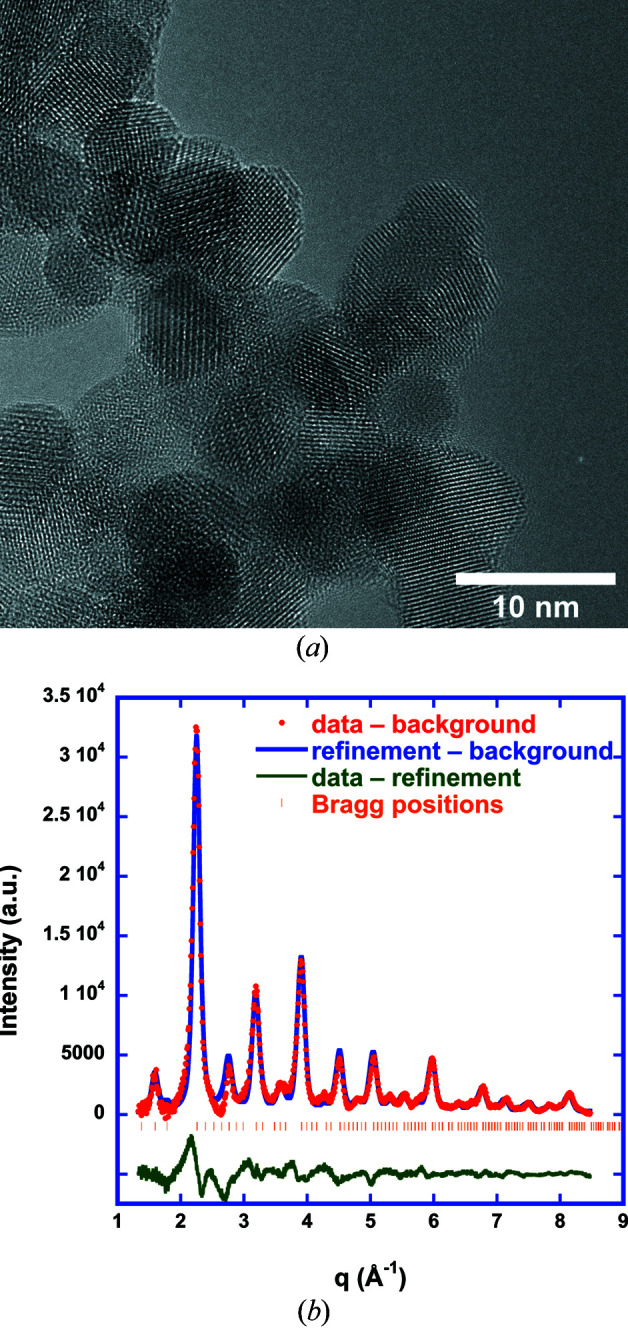
HRTEM image of LaFeO_3_ prepared by CVS (*a*) and Rietveld refinement of corresponding WAXS data (*b*).

**Figure 2:**
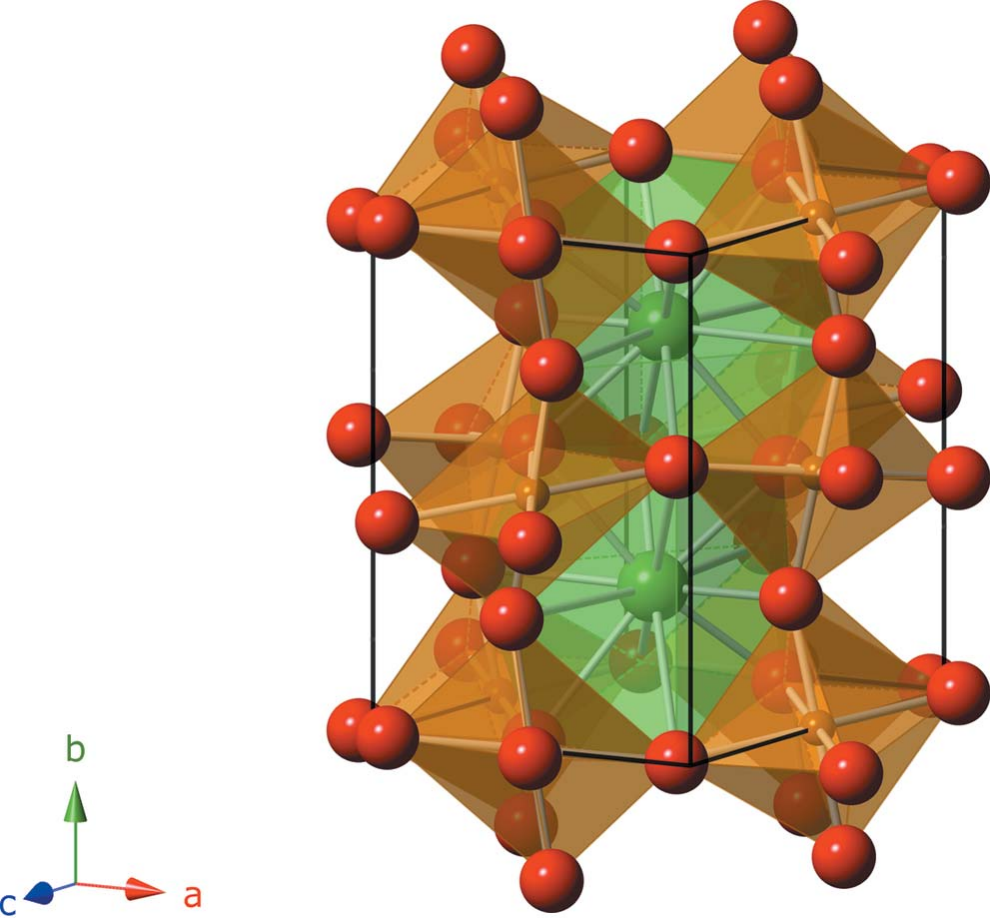
Unit cell of LaFeO_3_ displaying the coordination polyhedra for Fe–O (distorted octahedra, brown) and La–O (highly distorted icosahedra, green).

The nanocrystalline samples of LaFeO_3_ have been generated using chemical vapour synthesis (CVS) [analogous to the work of Stijepovic *et al.* (2015 ▸)] and are – despite being very small – highly crystalline, as is obvious from the high-resolution transmission electron microscopy (HRTEM) image (Fig. 1 ▸). The high crystallinity is confirmed in the X-ray scattering data by well defined Bragg reflections (Fig. 1 ▸) which are analysed using Rietveld refinement (Table 1 ▸) starting from data of Marezio & Dernier (1971 ▸) [Inorganic Crystal Structure Database (ICSD) code 28255] for twinned single crystals. LaFeO_3_ is a highly disordered perovskite with an orthorhombic lattice (space group *Pnma*, No. 62, Fig. 2 ▸). Fe is coordinated octahedrally to six O atoms at distances between 2.00 and 2.03 Å, while La has an extremely wide distribution of coordination distances to O: six at distances between 2.33 and 2.64 Å, and another six at distances between 2.80 and 3.09 Å. From line broadening of the diffraction pattern we determine a crystallite size of about 6 nm according to the Rietveld refinement, which is consistent with the TEM image (Fig. 1 ▸). The structural information from the Rietveld refinement is used to generate a physical model, *i.e.* a configuration of atoms, for further analysis by RMC.

**Figure 3:**
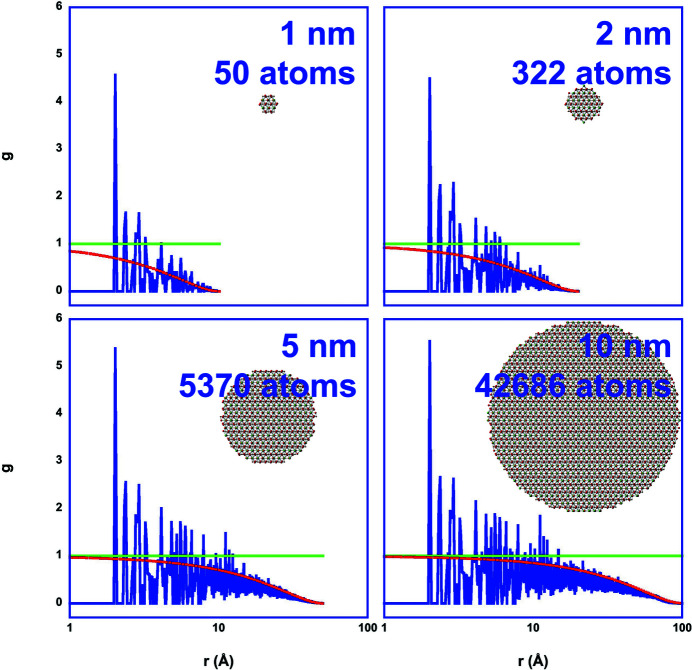
Finite size effect in the total PDF *g*(*r*) (blue) for LaFeO_3_ nanocrystals of 1–10 nm size. The total PDF is displayed together with the atom configuration, the corresponding envelope function assuming spherical clusters (red) describing the finite size effect in *g*(*r*) and the asymptotic value for infinitely large systems (green).

**Figure 4:**
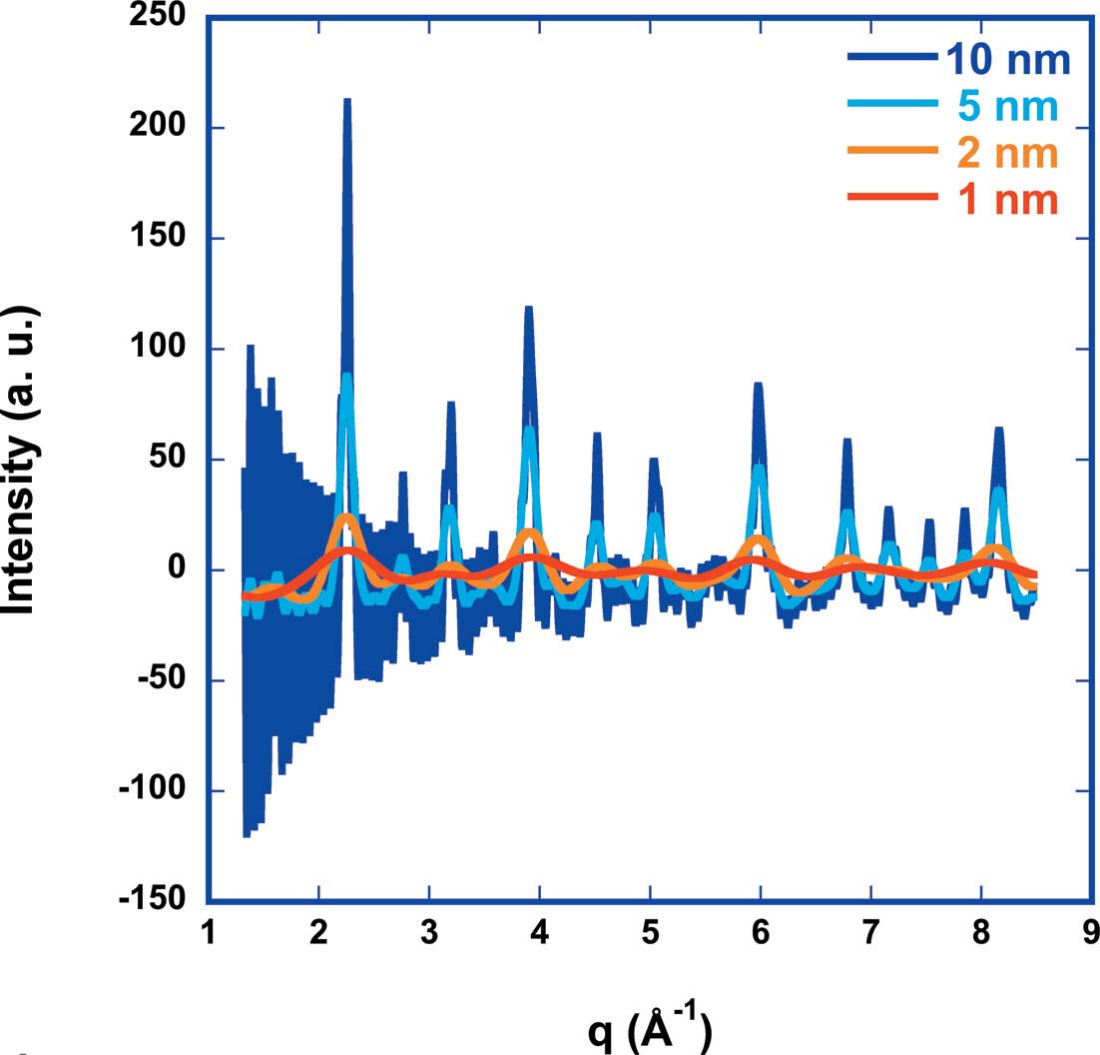
Computed scattering intensity for LaFeO_3_ of different crystal diameters using the structure-factor approach [equations (5)–(7) [Disp-formula fd7]].

Fig. 3 ▸ shows the effect of finite size on the total PDF for LaFeO_3_ for different particle (cluster) sizes. Typically, EXAFS may be able to discover structural information up to about 10 Å or 1 nm. However, for disordered systems only the first one or two coordination shells are observable, *i.e.* local structure information up to about 3–5 Å. Therefore, the finite size effect in pPDFs, which is described here using the shape function for spherical particles (Howell *et al.*, 2006 ▸; Gilbert, 2008 ▸) of diameter *d*,

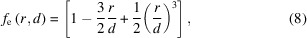

acts mostly as a reduction factor to the coordination number in the case of EXAFS (as displayed by the red curve) if no size-driven phase transition occurs. Scattering can detect much larger interatomic distances. However, the scattering intensity computed from pPDFs via the structure factor [equations (5)–(7) [Disp-formula fd7]] is distorted (Fig. 4 ▸). Clearly, large oscillations at small *q* values in reciprocal space are visible, which originate from the finite size of the atom configuration. Numerically, this happens because *g*(*r*) decays to 0 instead of approaching the asymptotic value of 1 for infinite systems. This adds unphysical contributions to the integral of the sinc function in *S*_*ij*_(*q*), which prohibits the use of this approach for refinement of scattering data of small nanoparticles.

The scattering intensity for isotropic samples may also be computed using the DSE instead of the structure factor:

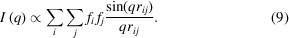

However, a direct implementation of the DSE is computationally too expensive for refinement of experimental data. Realizing that the numerator in the definition of the pPDFs [equation (3)],

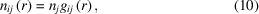

is the number of atoms of type *j* at a distance *r* from atom type *i*, we can use this information to compute the scattering intensity from a binned version of the DSE from *g*_*ij*_(*r*) efficiently:

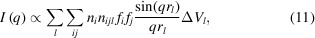

with the binned number of atom pairs

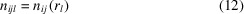

and the volume of a spherical shell of the width of a bin

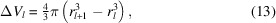

where *l* is the bin number assigned to the distance *r_l_* in the binned PDF. A speed-up regarding CPU time of a factor of 725 (1404) is observed for a 5 nm LaFeO_3_ particle containing 5378 atoms (6 nm, 9218 atoms), comparing the fast code using a bin width of 0.1 Å with a code using the DSE exactly.

**Figure 5:**
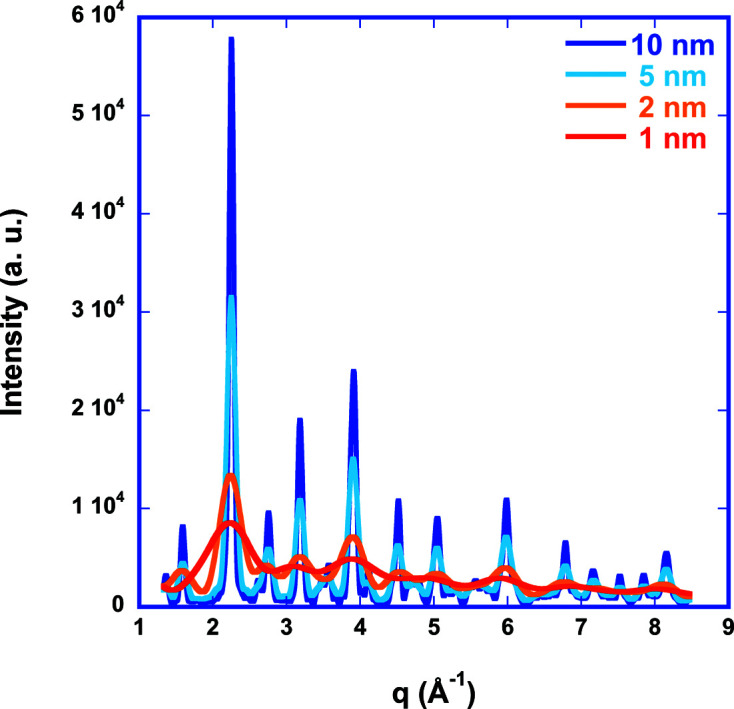
Computed scattering intensity for LaFeO_3_ of different crystal diameters using the binned DSE approach [equation (11)].

As shown in Fig. 5 ▸, we are now able to obtain scattering intensity data computed from cluster models for small nanocrystals without artefacts due to their finite size. This computation requires PDF bins up to distances larger than the cluster diameter. A comparison with a simulation using the exact DSE shows no significant deviations provided the bin width is between 0.1 and 0.01 Å. At 0.1 Å, some distortions are observed. For a bin width of 0.01 Å, the results are essentially equivalent to the exact computation of the DSE (compare Fig. S3 in the supporting information).

**Figure 6:**
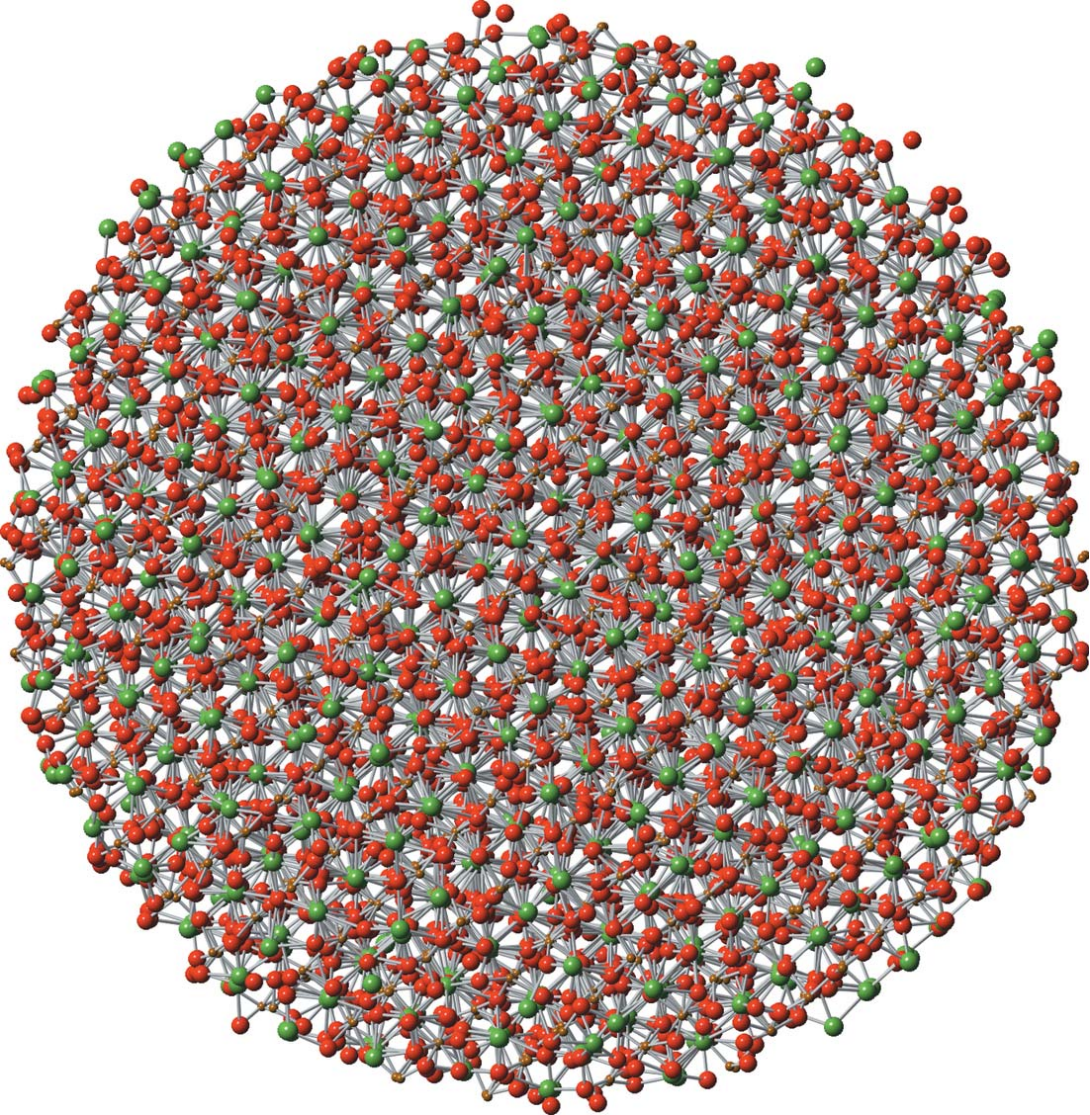
Atom configuration (60 Å diameter, 9218 atoms) after refining La *K* and Fe *K* EXAFS spectra together with WAXS data simultaneously (Fig. 7 ▸).

The described, computationally efficient method enables the simultaneous refinement of (X-ray, electron and neutron) scattering data and EXAFS spectra with one mutual physical model (Fig. 6 ▸), where the initial cluster model is generated from the results of the Rietveld refinement regarding crystallography and microstructure.

**Figure 7:**
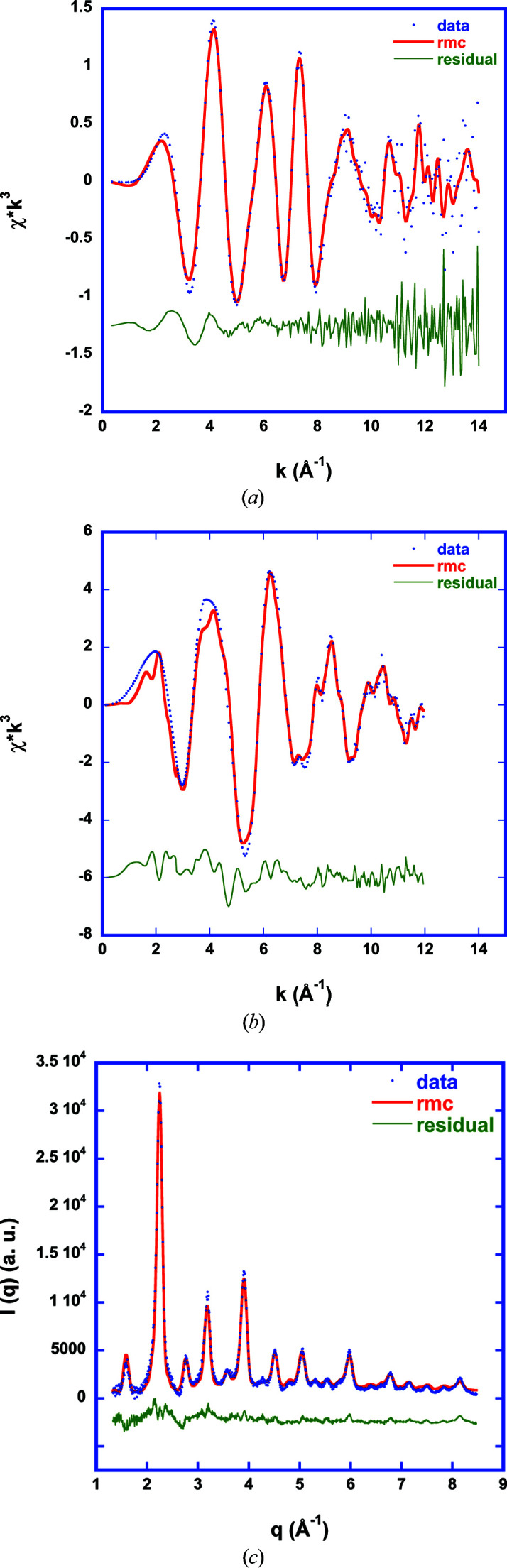
La *K* (*a*) and Fe *K* (*b*) EXAFS spectra fitted simultaneously with WAXS data (*c*) using a cluster model (Fig. 6 ▸).

Fig. 7 ▸ shows three data sets (La *K* and Fe *K* EXAFS spectra and WAXS data) fitted simultaneously with this cluster model algorithm. The differences between experimental data and refinement in the case of the Fe *K* spectrum between *k* = 2 Å^−1^ and *k* = 4 Å^−1^ are due to insufficient background subtraction (low-frequency signal in the residuum) and sharp X-ray absorption near-edge structure features which are not refined in RMC as it is computationally too expensive. The (high-frequency) deviations around *k* = 8 Å^−1^ are very likely due to multiple scattering which is not included in the code.

**Figure 8:**
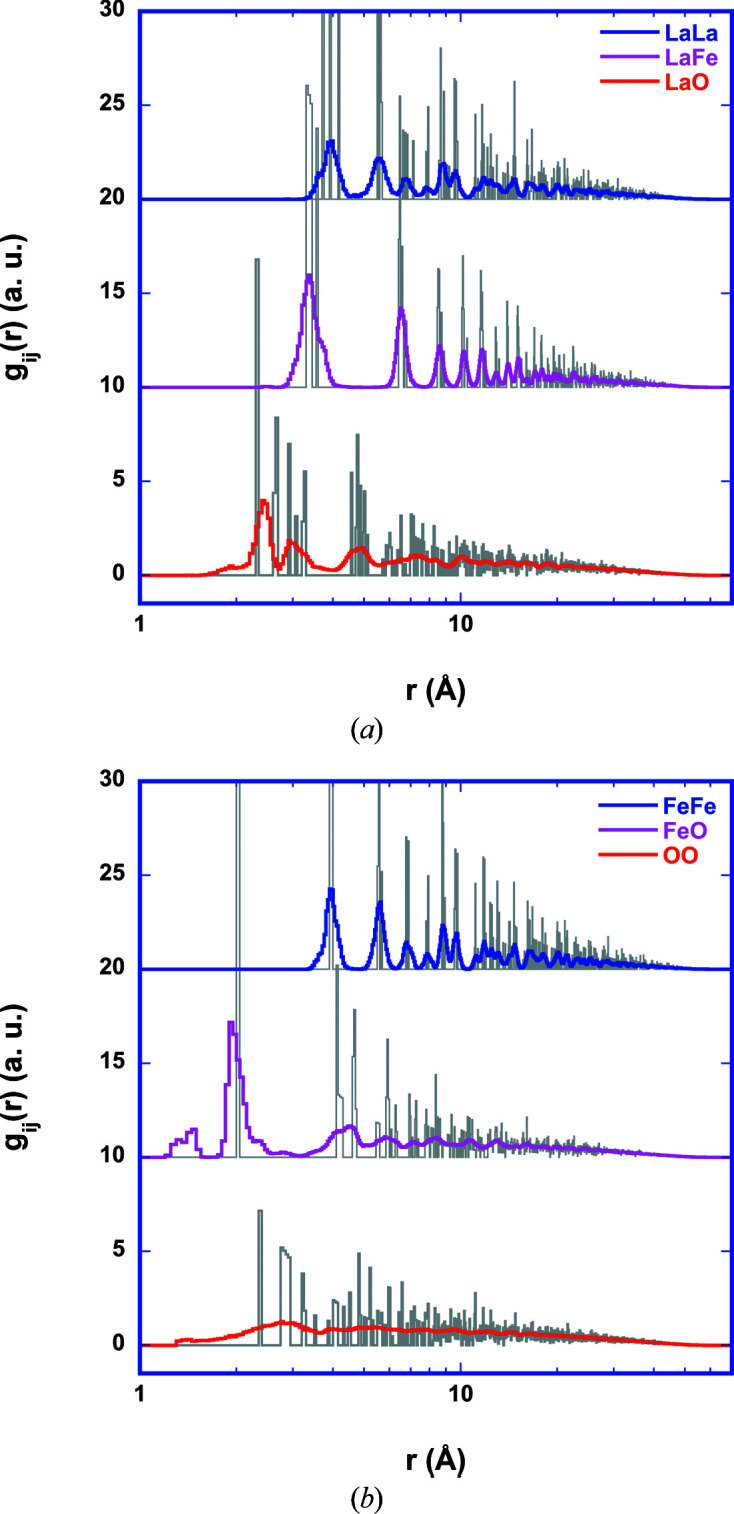
pPDFs as determined from simultaneous refinement of (*a*) La *K* and (*b*) Fe *K* EXAFS spectra of LaFeO_3_ together with WAXS (Fig. 7 ▸). Distribution functions computed from the initial atom configuration are displayed in grey.

The corresponding, refined pPDFs (Fig. 8 ▸) contain structural information which is consistent on the scale of the local structure and the long-range order. In the case of nanocrystalline LaFeO_3_, all pPDFs are essentially broadened versions of the distribution functions for the initial configuration. For Fe–O, an additional peak at about 1.4 Å is observed which is a numerical artefact since the hard-sphere radii chosen are slightly too small.

Since the small Fe–O peak is at a rather short distance, it contributes only about 0.5 O atoms to the total signal, which is smaller than or equal to the order of the typical error for determination of coordination numbers in RMC. Therefore, it may be neglected. Overall, the broadening of the pPDFs after the RMC refinement is caused by thermal and structural disorder. The obtained O–O correlation seems too broad. This is caused by the lack of direct information in EXAFS and the small atomic form factor of O compared with La and Fe (see Fig. S2 in the supporting information) in X-ray scattering. The inclusion of additional information regarding O through O *K*-edge spectra or neutron scattering data could help to remove this ambiguity. A closer look at the results of the moment analysis of the first peaks in the pPDFs shows that the coordination numbers and distances for the cation–cation distributions and the Fe–O distribution agree within the error estimate with the single-crystal data of Marezio & Dernier (1971 ▸). The corresponding coordination numbers and distances indicate that the nanoscaled LaFeO_3_ generated by CVS is highly crystalline and that local and long-range order are consistent. The refined La–O pPDF exhibits significant differences compared with the bulk material (Table 2 ▸). The difference corresponds to a reshuffling of one O atom between the first La–O peak at 2.36 Å and the second La–O peak at 3.18 Å. The total La–O coordination number of 11.5 agrees with the single-crystal result of 12 within the error estimate, especially when considering that the finite size effect already lowers the total coordination number to 11.1. The first peak in the La–O pPDF is shifted to shorter and the second peak to larger distances compared with the single-crystal data. Part of this shift in the peak is already observed in the initial configuration generated from Rietveld refinement of X-ray diffraction data, which may be explained by relaxation or reconstruction at the particle surface typically observed for oxides (see *e.g.* Diehm *et al.*, 2012 ▸). The additional shift could be due to reconstructions of the La–O coordination after the formation of La–(OH) groups at the particle surface following exposure to water vapour during storage in air or during the synthesis. In La(OH)_3_, La is coordinated to three O atoms at 2.57 Å and six O atoms at 2.76 Å (ICSD code 31584), and in LaOOH La is coordinated to six O atoms at distances between 2.36 and 2.63 Å (ICSD code 60675). Lanthanum oxide is hygroscopic (Gangwar *et al.*, 2017 ▸) and reacts with water vapour to form lanthanum hydroxide. The corresponding La–O bond length is longer than that in La_2_O_3_. Since the surface-to-volume ratio is orders of magnitude different for the small LaFeO_3_ particles compared with a single crystal, we speculate that La at the LaFeO_3_ surface is terminated by hydroxyl groups responsible for the additional long La–O coordination in the second La–O shell. A corresponding observation is not made for the Fe–O coordination.

This could mean that the LaFeO_3_ nanoparticles are terminated by La–O(H), which may be of high relevance to heterogeneous catalysis as the reactants interact via the surface with the catalyst.

## 4. Related literature

The following additional reference is cited in the supporting information: Grosse-Kunstleve (1992 ▸).

## 5. Conclusion

A solution to circumvent the finite size effect in RMC refinement of scattering data is the use of the DSE, which can be made computationally efficient through using the binned number of atom pairs via the pPDFs. Simultaneous analysis of several ‘raw’ EXAFS spectra and scattering data sets using a mutual physical model is enabled and allows in principle direct extraction of information for all pPDFs. The structural information obtained in this way is consistent regarding local structure and long-range order. Small nanoparticles are ideal candidates for this type of analysis where raw X-ray scattering and EXAFS spectra are available, since the line shape of the scattering data is dominated by the sample (microstructure, size and strain) and not by the instrument.

## Supplementary Material

Supporting information. DOI: 10.1107/S1600576722010858/jl5055sup1.pdf

## Figures and Tables

**Table 1:** Results of Rietveld refinement using space group *Pnma*, No. 62 *W*, Wyckoff position and multiplicity; *a*, *b*, *c*, lattice constants; *x*, *y*, *z*, fractional coordinates; *B*, displacement parameters; *d*, coherent diffracting domain size; ε, microstrain.

	*W*	*a* (Å), *x*	*b* (Å), *y*	*c* (Å), *z*	*B* (Å^2^)
Lattice constants		5.54 (4)	7.83 (6)	5.66 (4)	

La	4*c*	0.9939	0.75	0.0284	0.987 (5)
Fe	4*b*	0.0000	0.0000	0.5	0.477 (6)
O1	4*c*	0.0731	0.75	0.4875	1.167 (5)
O2	8*d*	0.7191	0.0393	0.2815	3.273 (6)
*d* (nm)		6.4 (1)			
ε (%)		0.68 (4)			

**Table 2:** Results of moment analysis of the first peaks in the pPDFs of LaFeO_3_ (Fig. 8 ▸) Numbers in bold are the results of the refinement, numbers in roman font are computed from the initial cluster configuration, and numbers in italics indicate coordination numbers and distances from the ICSD data for a single crystal.

Shell	Range (Å)	*N*	〈*r*〉 (Å)	*p*_2_ (Å^2^)
La–O	1.5–2.7	**4.6 (9)**	**2.36 (4)**	**0.04 (2)**
		5.6	2.47	
		*6*	*2.54*	
	2.7–4.0	**6.9 (13)**	**3.18 (6)**	**0.09 (3)**
		5.5	3.09	
		*6*	*3.06*	
Fe–O	1.7–2.7	**5.0 (10)**	**2.05 (4)**	**0.027 (12)**
		5.7	2.02	
		*6*	*2.01*	
La–La	3.0–4.5	**5.2 (9)**	**3.92 (4)**	**0.051 (12)**
		5.4	3.93	
		*6.0*	*3.94*	
La–Fe	2.7–4.5	**7.3 (11)**	**3.42 (4)**	**0.054 (14)**
		7.3	3.41	
		*8*	*3.41*	
Fe–Fe	3.0–4.8	**5.4 (8)**	**3.95 (3)**	**0.033 (9)**
		5.4	3.95	
		*6*	*3.93*	
O–O	1.5–3.7	**8.5 (12)**	**2.73 (8)**	**0.29 (5)**
		8.6	3.03	
		*8*	*2.99*	
