# Supplementary material for: Combining reverse Monte Carlo analysis of X-ray scattering and extended X-ray absorption fine structure spectra of very small nanoparticles
Source: J Appl Crystallogr. 2023 Feb 1;56(Pt 1):103–9. doi: 10.1107/S1600576722010858 (PMC9901930; doi:10.1107/S1600576722010858)
Supplement: Supplementary file 1 [file j-56-00103-sup1.pdf]

JOURNAL OF
APPLIED
CRYSTALLOGRAPHY

Volume 56 (2023)

Supporting information for article:

Combining Reverse Monte Carlo Analysis of X-ray Scattering and Extended X-ray Absorption Fine Structure Spectra of Very Small Nanoparticles

Markus Winterer and Jeremias Geiß

S1. EXAFS amplitude and phase function for La- and Fe-K spectra of $LaFeO_3$

The photoelectron wave vector

$$k = \left(\frac{2m\left(E - E_0\right)}{\hbar^2} \right)^{\frac{1}{2}}$$

is computed from X-ray photon energy E and energy of absorption edge E_0. Figure S1 compiles the amplitude and phase function for La- and Fe-K spectra of $LaFeO_3$ as computed from feff9 (Rehr et al. 2010) using the results of the Rietveld refinement of $LaFeO_3$.

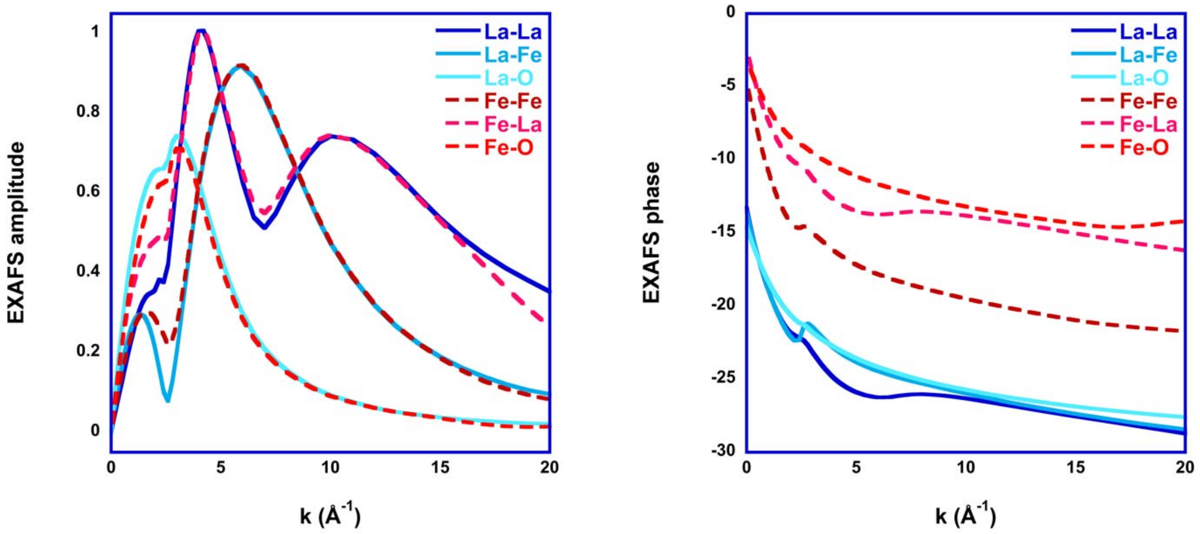

Figure S1 EXAFS single scattering amplitude $A(k,r)$ (left) and phase functions $\phi(k,r)$ (right) for La- und Fe-K-edge specta from feff9 (Rehr et al. 2010)

S2. X-ray atomic form factors

Figure S2 compiles the X-ray atom form factors for La, Fe and O (left) computed according to Grosse-Kunstleve (1992) and derived parameters for $LaFeO_3$ using the Cromer-Mann coefficients (a_1, b_1, a_2, b_2, a_3, b_3, a_4, b_4, c) for X-rays and electrons:

$$f_i(q) = c + \sum_{l=1}^{4} a_l \cdot \exp\{-b_l \cdot s\}$$

with

$$s = \frac{q^2}{16\pi^2}.$$

For neutron scattering one q-independent coefficient is used. The scattering vector is defined as

$$q = \frac{4\pi \sin\theta}{\lambda}.$$

The weighted average of the squared atomic form factors

$$\langle f^2 \rangle = \sum_i n_i f_i^2,$$

the squared average of the weighted atomic form factors

$$\langle f \rangle^2 = \left(\sum_i n_i f_i \right)^2,$$

and the coefficient

$$\beta_{ij} = \frac{n_i n_j f_i f_j}{\langle f \rangle^2}$$

are used to compute the total structure factor and scattering intensity (see Fig. S2 and S3).

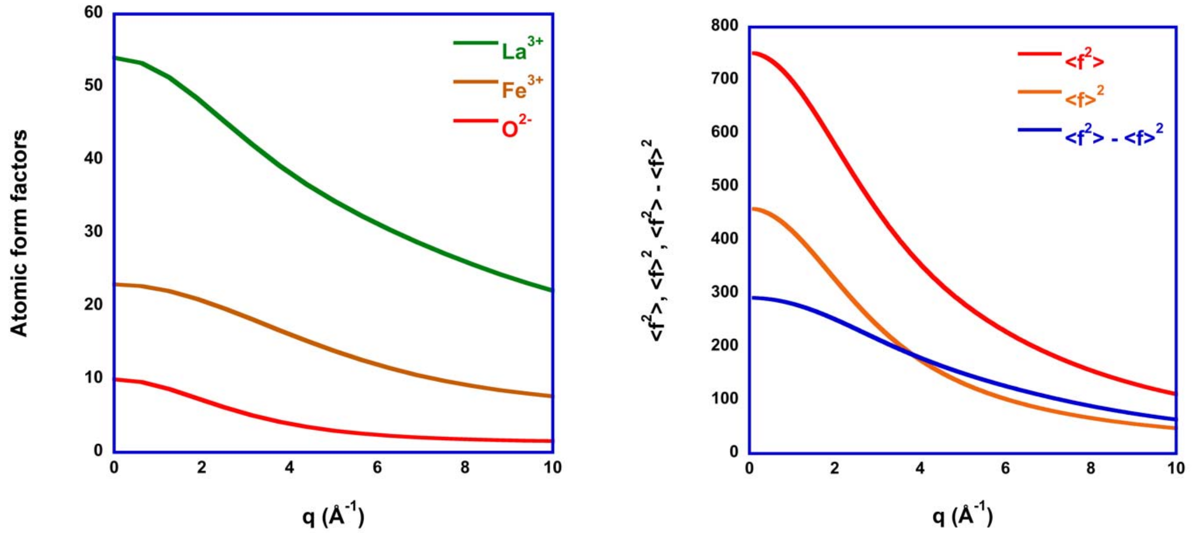

Figure S2 X-ray atomic form factors (left) and the average of the squared form factors, the squared average of the form factors and the difference of these two quantities (right)

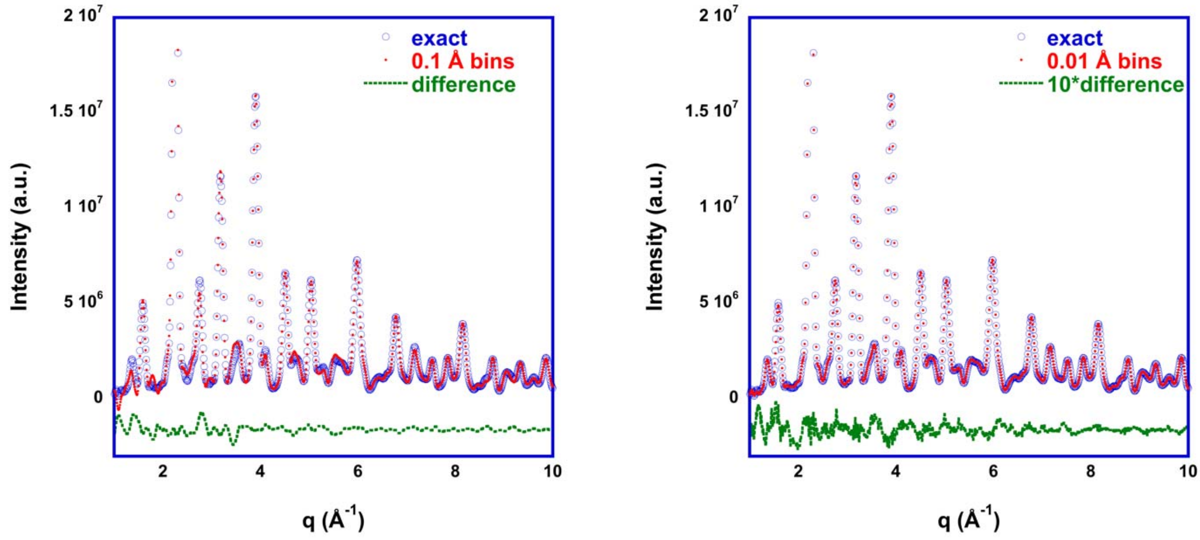

Figure S3 Comparison of scattering intensity computed via the exact Debye scattering equation and via the fast, binned version (using a bin width of 0.1 Å left and 0.01 Å right) from the initial atom configuration (please note the factor ten in the difference plot of the right figure).

References

R. Grosse-Kunstleve, in International Tables for Crystallography, Volume C, Mathematical, Physical and Chemical Tables, A. J. C. Wilson (ed.), Kluwer Academic Publishers Dordrecht Boston London 1992

Rehr, J. J., Kas, J. J., Vila, F. D., Prange, M. P., and Jorissen, K., Parameter-free calculations of x-ray spectra with FEFF9, Phys. Chem. Chem. Phys. 12 (2010) 5503-5513. doi.org/10.1039/B926434E
